# The integrated curriculum and student empathy: a longitudinal multi-cohort analysis

**DOI:** 10.1007/s10459-023-10292-1

**Published:** 2023-11-09

**Authors:** Christiane R. Herber-Valdez, Julie A. Blow, Tammy T. Salazar, Kathryn V. Horn, Dyanne G. Herrera, Naomi L. Lacy, Lisa Beinhoff, J. Manuel de la Rosa

**Affiliations:** 1 Office of Academic Affairs, Texas Tech University Health Sciences Center at El Paso, 5001 El Paso Drive, El Paso, TX 79905 USA; 2 Office of Institutional Research and Effectiveness, Texas Tech University Health Sciences Center at El Paso, El Paso, TX USA; 3 Department of Family Medicine, Paul L. Foster School of Medicine, Office of Academic Support, Texas Tech University Health Sciences Center at El Paso, El Paso, TX USA; 4 The University of Texas at El Paso, El Paso, TX USA; 5 Paul L. Foster School of Medicine, Office of Student Services, Texas Tech University Health Sciences Center at El Paso, El Paso, TX USA; 6 University of Houston College of Medicine, Houston, TX USA; 7 Texas Department of Health, Austin, TX USA; 8 Department of Medical Education, Paul L. Foster School of Medicine, Texas Tech University Health Sciences Center at El Paso, El Paso, TX USA; 9 Libraries of the Health Sciences, Texas Tech University Health Sciences Center at El Paso, El Paso, USA; 10 Department of Pediatrics Paul L. Foster School of Medicine, Office of Outreach and Community Engagement, Texas Tech University Health Sciences Center at El Paso, El Paso, TX USA

**Keywords:** Curriculum, Clinical training, Empathy, Medical education, Medical students, Student-patient interaction

## Abstract

Research has demonstrated erosion of empathy in students during medical education. Particularly, U.S. studies have demonstrated empathy declines during clinical training in the third and fourth year of traditional medical programs. Yet, studies conducted outside the U.S. have not confirmed this trend. Timing and extent of patient interactions have been identified as empathy-protective factors. The need to examine empathy within different learning contexts has been noted, as has the need for longitudinal and time-series research designs to analyze trajectories. Between fall 2010 and spring 2019, we assessed empathy longitudinally among six student cohorts (N = 493) at a U.S. medical school, where patient interaction occurs early and throughout an integrated curriculum. Empathy levels of students in each cohort were assessed at five time points utilizing the Jefferson Scale of Physician Empathy-Student version. We hypothesized empathy levels will not degrade by program end, and trajectories will not show patterns of decline in Years Three and Four. Analysis of Variance (ANOVA) and Linear Mixed Model (LMM) analyses were used to analyze differences at baseline and changes in empathy trajectories. ANOVA analyses revealed statistically significant differences at baseline by class cohort (*F*(5, 487) = [23.28], *p* < 0.001). LMM analyses indicated empathy was either significantly higher or not different at the end of the program (*F*(19, 1676) = [13.97], *p* < 0.001). Empathy trajectories varied among cohorts; yet, none resulted in an overall empathy decline by the end of the program. Findings demonstrate empathy in U.S. medical students can be unchanged or higher by the end of medical education. Outcomes are consistent with reports of non-declining medical student empathy outside the U.S. and support the notion of context-specificity. Results further support recent research, suggesting decreases in empathy during training can stabilize or increase by program end. These findings have important implications for future empathy research context and design considerations, as well as program planning.

## Introduction

In the context of medical education, empathy has been defined as a “predominantly cognitive attribute that involves an understanding of the patient’s experiences, concerns, and perspectives, combined with a capacity to communicate this understanding and an intention to help” (Hojat, 2016, p. 74). Empathy is generally accepted as a fundamental part of the doctor-patient relationship (Pedersen, 2009), and has been considered “one of the most highly desirable professional traits that medical education should promote” (Newton et al., 2008, p. 244). Among patients, empathy results in higher satisfaction, increased adherence to treatment plans, improved clinical outcomes, and reduced emotional distress. In physicians, empathy is associated with increased diagnostic accuracy, lower burn-out, higher well-being, higher clinical competence ratings, and reduced medical-legal risk (Bellini & Shea, 2005; Kelm et al., 2014; Neumann et al., 2011). Given its impact on medical practice, the American Association of Medical Colleges (AAMC) asserts “physicians must be compassionate and empathetic in caring for patients,” and includes empathy among the educational objectives for undergraduate medical education (The Medical School Objectives Writing Group, 1999, p. 15). Consequently, medical schools aim to produce physicians who are not only knowledgeable and skilled, but also empathic.

Unfortunately, examinations of empathy in medical students have demonstrated inconsistent and contradictory patterns (Spatoula et al., 2019), with many studies reporting students’ empathy to be stunted during medical school (Pedersen, 2009). U.S.-based studies, in particular, have demonstrated medical students’ empathy to decrease, as they progress through medical education (Austin et al., 2007; Bellini & Shea, 2005; Chen et al., 2007; Chen et al., 2012; Hojat et al., 2004; Neumann et al., 2011). Despite some challenges to reported methodologies (Colliver et al., 2010b), empathy decline has been accepted as an established trend among medical educators and researchers.

Empathy decline has been reported to exist regardless of baseline empathy levels, and has been linked to the onset of patient exposure during clinical training, which traditionally occurs in the third and fourth year of U.S. medical school programs (Austin et al., 2007; Chen et al., 2007, 2012; Hojat et al., 2009; Hojat et al., 2009). A systematic review of research spanning a 10-year period (Neuman et al., 2011) revealed significant decreases, with most studies showing the decline during the third and fourth year of the medical education curriculum. The authors suggest programmatic inadequacies related to student-patient interactions in clinical years, such as (1) short length of time with patients, resulting in fragmented patient-physician relationships, (2) unsuitable learning environments, including few bedside interactions, and (3) inadequate role models and an idealized view of the medical profession, as potential causes of empathy decline (Neumann et al., 2011).

Demographic factors, such as gender and age, have also been associated with empathy. Here, the majority of studies indicate females maintain greater levels of empathy throughout training, relative to males (Austin et al., 2007; Chen et al., 2012; Duarte et al., 2016; Hegazi & Wilson, 2013; Kataoka et al., 2009; Magalhães et al., 2011; Silva et al., 2014; Montilva et al., 2015; Wen et al., 2013). With respect to age, studies of programs that enroll students directly after high school, suggest younger students lack experience in interpersonal interactions, and empathy is subsequently bolstered throughout medical training (Kataoka et al., 2009; Roh et al., 2010; Wen et al., 2013).

While many U.S. studies have identified empathy declines during medical programs, much of the research conducted outside of the U.S. has not confirmed this trend. Roff (2015) compared empathy scores from 18 studies conducted between 1990 and 2010, and found most non-US studies reporting a trend towards incline in empathy, rather than decline. The author points out that clinical exposure occurs at different points of the curriculum in different countries, and that the third year is different in a four-year U.S. curriculum versus programs in other countries. A scoping review of 20 multi-national studies conducted between 2009 and 2016 (Ferreira-Valente et al., 2016) also did not confirm empathy decline as a generalizable international trend, with many non-U.S. studies reporting positive or no changes in empathy, while included U.S. studies observed decreases in empathy (Chen et al., 2012; Hojat et al., 2009). A recent analysis of 40 multi-national empathy studies (Ponnamperuma et al., 2019) suggests context-based patterns, demonstrating similarities in the results of studies from the same geographical regions, with U.S. studies reporting mostly negative changes. The authors propose an “emerging geo-sociocultural pattern in the change in empathy” (Ponnamperuma et al., 2019, p. 6).

Though differences in research designs, methodological frameworks, and empathy instruments need to be considered, these outcomes support the concept of context specificity, emphasizing the need to better understand educational settings, curricula, and program conditions that nurture empathy during medical training (Eva, 2003; Quince et al., 2016). Consequently, researchers have called for examinations of empathy within different curricular environments and for exploration of unique characteristics within medical schools (Ferreira-Valente et al., 2016; Neumann et al., 2011; Ponnamperuma et al., 2019; Quince et al., 2016).

Literature in this area is still scarce; however, initial studies have identified curricular structures and local learning contexts as potential factors accounting for varied empathy findings. Empathy-enhancing educational approaches have been found to yield stable empathy scores, challenging “the idea that declines in empathy previously reported in longitudinal US studies are generalizable to all medical schools either in the USA or elsewhere” (Costa et al., 2013, p.520). Notably, research has suggested early exposure to clinical training could be related to higher empathy scores in later years of medical school (Kataoka et al., 2009; Roh et al., 2010; Wen et al., 2013), pointing to the extent and timing of patient-interaction as critical factors in preserving empathy.

Dornan et al. (2006, p.3) note early experiences help students develop empathic reactions towards patients, and “makes their learning more real and relevant”. Krishnasamy et al., (2019, p. 1223) add that “in order to show empathy and compassion to patients, medical students need to develop and maintain perspective of seeing the patient as a person over the course of their medical training”*.* The authors stress that empathy and compassion are dynamic processes that are relational in nature, and that the interactions between the patient, medical student, and training environment all affect medical students’ learning of empathy and compassion.

### Curriculum reform at U.S. Medical Schools

Medical education in the U.S. has seen a philosophical shift and a call for reform of traditional training programs, noting the importance of patient interactions and embedding patient experiences into the early phases of medical education programs. The traditional ‘‘2 × 2’’ curricular structure, in which 2 years of basic science are followed by 2 years of clinical science, is widely considered inadequate in preparing future physicians (Cooke et al., 2006; Irby et al., 2010). Landmark publications, such as the Carnegie Foundation’s “Educating Physicians: A Call for Reform of Medical School and Residency” (Cooke et al., 2006; Irby et al., 2010) have called for early clinical immersion to help students integrate skills and knowledge in preparation for practice. Consequently, the medical education community has increasingly embraced the value of early patient contact experiences for medical training (Wenrich et al., 2013), and the ‘‘integrated curriculum has rapidly risen to popularity” (Brauer & Ferguson, 2015).

The *integrated curriculum* typically represents curricular innovations, such as embedding ethics and clinical skills into first-year courses; integrating basic science courses with preclinical or clinical courses; and integrating clinical exposure into early phases of medical education programs (Brunger & Duke, 2012; Dyrbye et al., 2011; Klement et al., 2011; Ogur et al., 2007; Radwany et al., 2011; Schwartz et al., 1999; Yu et al., 2009; as cited in Brauer & Ferguson, 2015, p.313). Early clinical experience has been defined as pre-clerkship experiences with authentic patient contact to enhance learning of health, illness or disease, and the role of the health professional (Cooke et al.,^2010^; Dornan et al., 2006; Yardley et al., 2010), with ‘early’ referring to the first two years of medical education or ‘pre- clinical’ phase (Littlewood et al., 2005; Yardley et al., 2010). Early experiences help students develop appropriate attitudes towards their studies, while reducing the “shock of practice” students might experience as they enter the clerkship phase (Diemers et al., 2008; Godefrooij et al., 2010, p. 1; Littlewood et al., 2005).

While the literature on perceived benefits of early experiences is strong (Dornan et al., 2006; Yardley et al., 2010), Yardely et al. (2010) note there are still questions about how early experiences lead to particular outcomes. Although numerous studies suggest enhancing patient interactions positively impacts student attitudes toward patient care, examinations of student empathy levels in those learning environments are scarce. Therefore, it remains unclear whether or how empathy changes, as medical students progress through integrated medical education programs. A recent study assessing the educational environment in an integrated curriculum in the United Arab Emirates found students’ awareness of empathy as a persistent strength over the entire course of study (Shehnaz, 2019). However, we did not find studies that systematically assess student empathy levels within the context of integrated curricula in the U.S. In addition, researchers have called for testing empathy interventions at multiple time points (Hojat, 2009) and for assessing empathy longitudinally (Piumatti et al., 2020).

Given this current state of the literature, our study examined empathy levels longitudinally at several time points among multiple cohorts of students enrolled at a U.S. medical school with an integrated curriculum, where student-patient interaction occurs early, and is embedded in all four years of the medical education program.

### Specific aims and hypotheses

Our specific aims were to (1) examine empathy levels longitudinally across six student cohorts, while controlling for age and gender; (2) examine empathy levels within each cohort at five time points over the course of the medical education program; and (3) examine differences in empathy levels by cohort and gender. We hypothesized that (1) students’ empathy levels will not decline by the end of the program, (2) empathy trajectories will not show patterns of decline in the third and fourth year of the program, and (3) female students will exhibit higher levels of empathy compared to male students.

## Methods

### Study setting: an integrated medical education program in the U.S.

Aligned with research on integration and early clinical experiences (Cooke et al., 2010), students at the Texas Tech University Health Sciences Center at El Paso (TTUHSC El Paso) Paul L. Foster School of Medicine are immersed in both basic and clinical sciences beginning in Year One of the four-year program. The course of study, designed around clinical presentations within organ system-based units, introduces students to clinical scenarios and delivers the corresponding basic science concepts simultaneously. Within this curricular model, students are exposed to patients starting in the first week and throughout the program.

In Years One and Two of the program, integration is achieved through the delivery of four interrelated courses, which span the pre-clerkship phase. The cornerstone of this phase is the *Scientific Principles of Medicine* (SPM) course organized around 70 plus clinical presentations, which are assigned to a corresponding organ system unit. Presentations are delivered in the form of clinical schemes, designed to instruct students in the relevant basic science content and specific pathophysiological processes associated with each presentation. Concurrently, students participate in the *Medical Skills* course, which incorporates interactions with standardized patients and simulations to prepare students for patient interviews and examinations. Students learn with patients presenting clinical problems that are simultaneously addressed in SPM. In addition, students engage in reflective writing exercises—reported to improve empathy (Chen & Forbes, 2014).

**Fig. 1:**
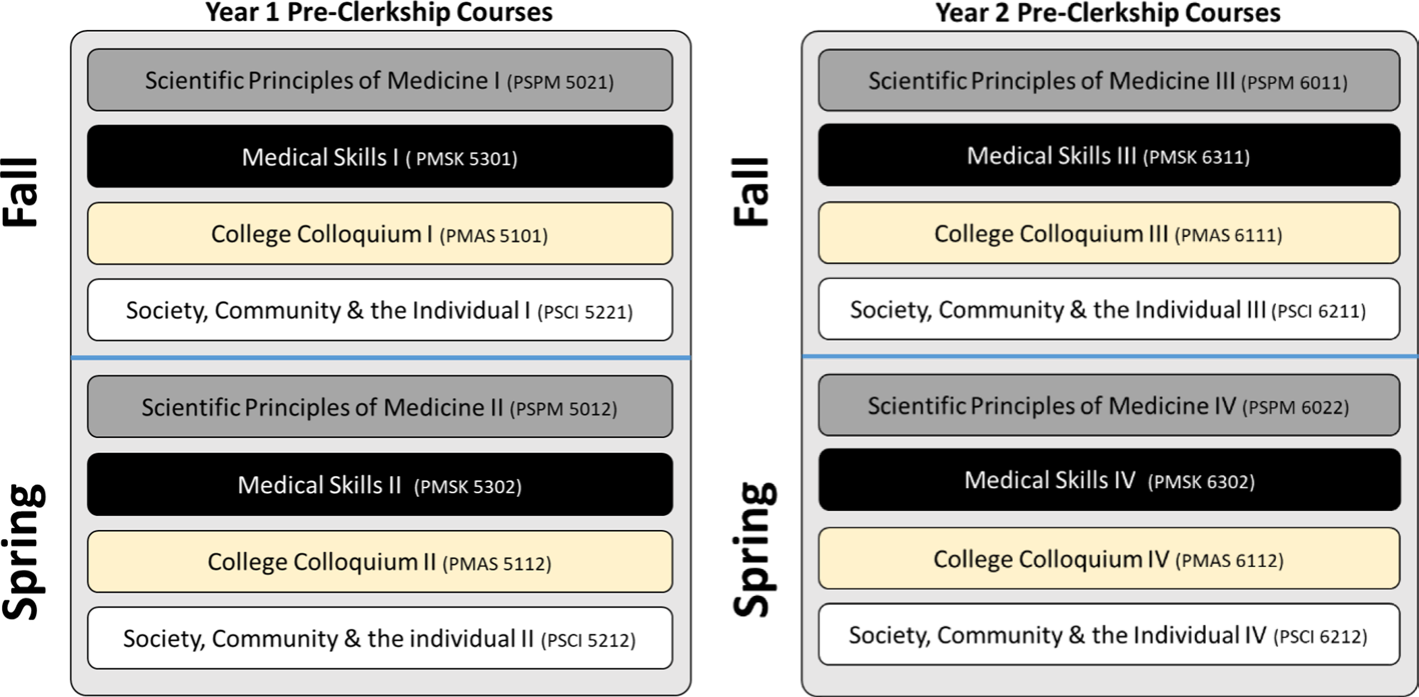
Interrelated Pre-Clerkship Courses

The *Society, Community and the Individual* (SCI) course immerses students in clinical experiences and empathy-related topics, such as patient-centered interviewing, cultural intelligence, and cultural awareness. Sessions utilize standardized patients, and upon course completion students interact with actual patients eight times over the course of a year and a half. The fourth course, *College Colloquium,* promotes critical reflection through faculty-led discussions on patient-centered and empathy-related topics. Guided reflections have been found to allow students “to pause and contemplate on their experiences of empathy and compassion” (Krishnasamy et al., 2019, p. 1228; Pedersen, 2009). Refer to Figure 1 for a depiction of Years One and Year Two pre- clerkship courses.

**Fig. 2:**
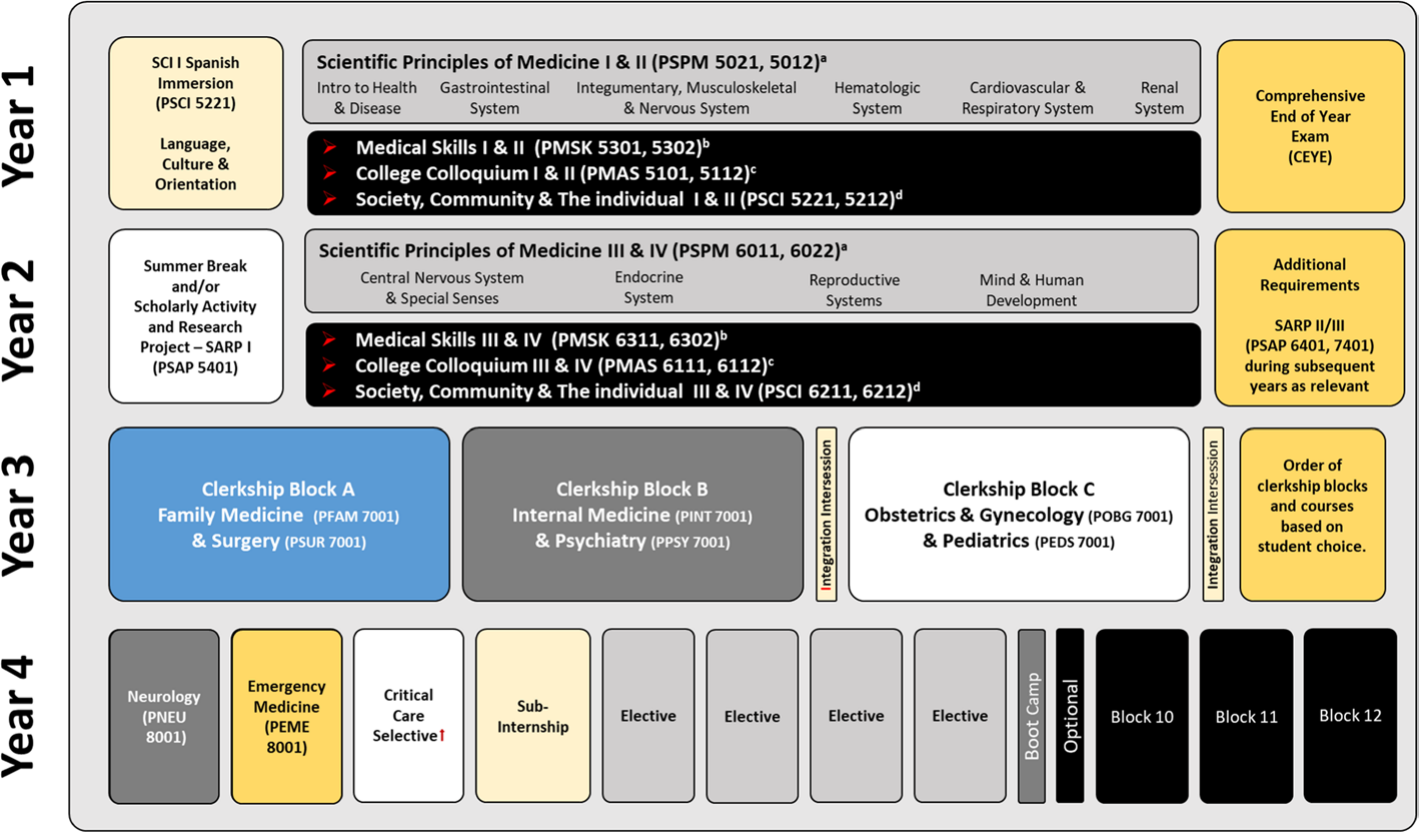
Four-Year Integrated Curriculum Overview. Source: Paul L. Foster School of Medicine Academic Catalog 2018–19. For current PLFSOM curriculum overview see https://elpaso.ttuhsc.edu/som/catalog/ContentOverview.aspx. *Notes* ^a^Scientific Principles of Medicine (SPM) is organized around clinical presentations, which are assigned to a corresponding organ system unit. Presentations are delivered in the form of clinical schemes, designed to instruct students in the relevant basic science content and presentation-specific pathophysiological processes. ^b^Medical Skills incorporates interactions with standardized patients and simulations to prepare students for patient interviews and examinations. Patients present clinical problems that are simultaneously addressed in SPM. ^c^College Colloquium promotes critical reflection through faculty-led discussions on patient-centered and empathy-related topics. ^d^Society Community and the Individual (SCI) immerses students in clinical experiences and empathy-related topics. Sessions utilize standardized patients and upon course completion students interact with actual patients

Concentrated clinical activities begin in Year Three, divided into three 16-week clerkship blocks. Each block consists of two clerkship disciplines with embedded longitudinal clinical experiences. Each block also contains didactic sessions on specialty-specific topics. Here, students reflect on common experiences, discuss ethical dilemmas, and participate in real-life simulations designed to prepare students for challenging physician-patient interactions. In addition, two intersessions integrate clinical rotation experiences with concepts from Year One and Year Two coursework. Year Four continues with required clinical block rotations, sub-internships and elective experiences. In addition, a two-week long *boot camp* delivers simulations and interactive learning modalities to prepare students for the transition from medical student to first year resident. Refer to Figure 2 for a diagram depicting the four-year curriculum.

Throughout the program, emphasis is placed on patients’ roles as key informants and *teachers*, who provide information needed for diagnoses and treatment. Focus is placed on developing positive physician-patient relationships that consider life circumstances and cultural perspectives. Communication is considered a central component of relationship-building, and students are required to complete a course in Medical Spanish to better serve the local Hispanic population. Through the program’s patient-centered curricular components, empathy is continually reinforced, and students are trained to learn from and with patients.

### Participants

Participants were 493 medical students [females, n = 213 (43.2%); males, n = 278 (56.4%); majority (67.5%) ≤ age 24] enrolled at the Paul L. Foster School of Medicine between Fall 2010 and Spring 2019. All medical students participate in annual empathy assessments during the course of the medical education program; therefore, no study recruitment was necessary. The sample included students from six cohorts (Class of 2014—Class of 2019), representing 93% of the total medical student population (529) matriculating over the span of six academic years (Academic Year 2010–11 to Academic Year 2015–16). Of the original 493 participants, two students who could not be matched to a unique research identifier across all four years were excluded, as were 19 students who repeated a program year. Table 1 shows the number of students per class cohort, as well as the age and gender distributions for each cohort. Students only had one opportunity to complete the empathy assessment at each time point; therefore, the number of participants within each cohort varies across time. The total count of students from each cohort who completed the assessment at each time point is included in Table 2.

**Table 1:** Selected characteristics of 493 medical students enrolled between 2010–2015 by Class Cohort

Characteristic	Students, No. (%)						
	Class of 2014	Class of 2015	Class of 2016	Class of 2017	Class of 2018	Class of 2019	All Cohorts
Students, No.	53	73	68	97	98	104	493
*Age*							
≤ 24 years	35 (66.0)	56 (76.8)	49 (72.1)	64 (66.0)	71 (72.4)	58 (55.8)	333
≥ 25 years	18 (34.0)	17 (23.3)	19 (27.9)	33 (34.0)	27 (27.5)	46 (44.2)	160
*Gender*^*a*^							
Male	29 (55.8)	39 (53.4)	38 (55.9)	62 (64.6)	43 (43.9)	67 (64.4)	278
Female	23 (44.2)	34 (46.6)	30 (44.1)	34 (35.4)	55 (56.1)	37 (35.6)	213

*No.* number

^a^Data on gender was missing for two students. Therefore, gender values do not add to total number of participating students in cohorts for Class of 2014 and 2017

**Table 2:** Means and standard deviations of JSPE-S scores of 493 medical students at five time points (Time 1–Time 5)^a^ by class Cohort

Class Cohort	Entering AY	Time 1^b^		Time 2		Time 3		Time 4		Time 5	
		No.	Mean (SD)	No.	Mean (SD)	No.	Mean (SD)	No.	Mean (SD)	No.	Mean (SD)
2014	2010–11	53	109.9	42	112.9*	52	101.0**	51	98.4**	32	106.8
			(11.6)		(11.4)		(13.6)		(14.7)		(11.1)
2015	2011–14	73	108.7	69	100.2*	71	105.0**	NA	NA	66	110.8*
			(13.5)		(12.4)		(13.2)				(13.5)
2016	2012–13	68	101.7	67	105.8	65	114.1**	59	114.8**	67	113.9**
			(7.8)		(11.6)		(10.7)		(11.9)		(13.6)
2017	2013–14	97	108.2	96	113.2	92	114.5**	69	114.3**	87	114.2**
			(6.3)		(12.0)		(13.9)		(12.3)		(14.3)
2018	2014–15	98	113.2	94	109.6*	84	113.0**	81	108.9**	83	111.7
			(9.4)		(11.3)		(9.9)		(12.5)		(11.4)
2019	2015–16	104	116.5	96	113.7*	88	113.4*	100	110.7*	60	113.6
			(8.4)		(10.2)		(12.7)		(12.4)		(12.7)
All Cohorts		493	110.3	464	109.5*	452	110.9*	360	109.9*	395	112.4
			(10.5)		(12.4)		(13.3)		(13.6)		(13.1)

*AY* academic year; *No.* number; *SD* standard deviation; *NA* not analyzed

*p < 0.05

**p ≤ 0.001

^a^The JSPE-S was administered to students at the beginning of each academic program year (Times 1–4) and at time of graduation (Time 5)

^b^Time 1 is used as the mean comparison group in the ANOVA analysis

### Instrument

We used the Jefferson Scale of Physician Empathy-Student version (JSPE-S) (Hojat et al., 2001, 2002, 2003) to measure medical students’ empathy levels. Among currently available empathy scales (e.g. Interpersonal Reactivity Index, Balanced Emotional Empathy Scale, Empathy Quotient, Questionnaire of Cognitive and Affective Empathy), only the JSPE-S was specifically developed to measure medical students’ orientation toward physician empathy in patient-care situations, making it an appropriate instrument for medical education students and practitioners of health professions (Hojat & Gonnella, 2017; Hojat et al., 2002).

The JSPE-S includes 20 Likert-type items which are answered on a seven-point scale (1 = ‘‘strongly disagree,’’ 7 = ‘‘strongly agree’’). Psychometric data supporting the construct validity and criterion-related validity (convergent and discriminate) of the JSPE-S have been reported, and internal consistency was adequate (α = 0.89). Overall, there is wide agreement that the JSPE is based on extensive research and has a solid psychometric foundation (Colliver et al., 2010a; Colliver et al., 2010b; Roff, 2015). Reliability of the scale for the current study was adequate (α = 0.84).

### Procedure

We utilized a longitudinal, repeated measures survey instrument approach, assessing empathy levels with the JSPE-S in six student cohorts, across the course of the four-year program. Our study was approved by the Institutional Review Board. All medical students within each class cohort complete the JSPE-S at five time points, as part of regular programmatic assessments. The JSPE-S is administered electronically utilizing the survey software Qualtrics (Qualtrics, Provo, Utah) at the beginning of Year One (Time 1), at the beginning of each subsequent program year—Years Two, Three and Four—(Times 2–4), and at the end of the program (Time 5). To maintain confidentiality, student information was de-identified and randomly generated identification numbers were used during data analyses. Transfer students and students who repeated the instrument at any time point were excluded. Due to attrition, not all students participated in all five surveys. Additionally, survey data for the Class of 2015 was inconsistently collected at Time 4, such that we could not obtain a complete data set for this time point. Therefore, Class of 2015 survey data for Time 4 was not included in the analysis.

We used SPSS Statistics Version 24.0 (IBM, Armonk, New York) for all statistical analyses. In order to test for baseline differences in empathy scores, an ANOVA was conducted to assess baseline scores among the cohorts, as well as by gender within each cohort, at Time 1. Tukey’s HSD test was used to compare individual means within the ANOVA. To test for significant differences in empathy over time (at five time points), we performed linear mixed model (LMM) analyses for repeated measures. Class cohort, time the JSPE-S was taken, age, gender, and an interaction variable of class cohort and time the JSPE-S was taken, were entered into the model as fixed effects, while the participants’ unique research identifiers were specified as a random effect. Since prior research indicates age and gender correlate with empathy (Austin et al., 2007; Bellini & Shea, 2005; Chen et al., 2007; Chen et al., 2012; Duarte et al., 2016; Hegazi & Wilson, 2013; Hojat, 2009; Hojat et al., 2004; Kataoka et al., 2009; Kelm et al., 2014; Magalhães et al., 2011; Montilva et al., 2015; Neumann et al., 2011; Roh et al., 2010; Silva et al., 2014; The Medical School Objectives Writing, 1999; Wen et al., 2013) we included age and gender as control variables in the analysis. We calculated effect size estimates to determine the magnitude of variance. Statistical significance was determined at *p *values less than 0.05. We conducted these analyses for each class cohort and for the combined cohorts. The following section describes the results of our analyses, organized by the presentation of student empathy levels exhibited at the beginning of the program, at program end, and across program years.

## Results

### Empathy at baseline

Individual cohort mean JSPE-S scores at baseline ranged from 101.7 (Class of 2016) to 116.5 (Class of 2019). The mean JSPE-S score for all cohorts combined was 110.3. Refer to Table 2 for a presentation of mean JSPE-S scores by class cohort and combined cohorts at baseline (Time 1). An ANOVA to assess baseline differences in empathy scores among the cohorts revealed statistically significant differences by class at Time 1 for all cohorts (*F*(5, 487) = [23, 28], *p* < 0.001). Tukey’s HSD test showed the Class of 2019 mean empathy score (116.5) was significantly higher at Time 1 (*p* < 0.05), in comparison to all other cohorts, with the exception of the class of 2018, where no statistically significant difference was found (*p* = 0.151). The Class of 2018 mean empathy score (113.2) was significantly higher at Time 1, in comparison to the Classes of 2015 (*p* = 0.024, 95% CI [0.36, 8.76]) and 2017 (*p* = 0.003, 95% CI [1.12, 8.90]). Means for the Classes of 2015 and 2017 were 108.7 and 108.2, respectively. In addition, all cohorts’ empathy scores were significantly higher at Time 1 (all *p*s <0 .05), in comparison to the Class of 2016, which had the lowest mean score of the six cohorts (101.7).

Results of this analysis are included in Table 3.

**Table 3:** Mean comparisons of JSPE-S scores of 493 medical students at baseline (Time 1) by Cohort

Class Cohort	Class comparison	Mean difference	Std. Error	Sig	(95% CI) Lower bound	(95% CI) Upper bound
2014	2015	1.258	1.713	0.978	− 3.64	6.16
	2016	8.267	1.739	< .001	3.29	13.24
	2017	1.706	1.622	0.900	− 2.93	6.35
	2018	− 3.302	1.619	0.321	− 7.93	1.33
	2019	− 6.537	1.602	0.001	− 11.12	− 1.95
2015	2016	7.008	1.6	< .001	2.43	11.59
	2017	0.448	1.471	1.000	− 3.76	4.66
	2018*	− 4.56	1.468	0.024	− 8.76	− 0.36
	2019	− 7.796	1.449	< .001	− 11.94	− 3.65
2016	2017	− 6.561	1.501	< .001	− 10.86	− 2.26
	2018	− 11.568	1.498	< .001	− 15.86	− 7.28
	2019	− 14.804	1.48	< .001	− 19.04	− 10.57
2017	2018*	− 5.008	1.36	0.003	− 8.9	− 1.12
	2019	− 8.244	1.34	< .001	− 12.08	− 4.41
2018	2019	− 3.236	1.336	0.151	− 7.06	0.59

The table shows results of an ANOVA comparing baseline scores across class cohorts

*Std.* standard; *Sig.* significance

*p < 0.05

ANOVAs were also used to assess baseline differences in empathy scores by gender for each cohort. These analyses revealed that empathy scores in females were significantly higher than males in the Class of 2014 (*F* (1, 50) = [8.25], *p* = 0.006, 95% CI [111.03, 118.97]), the Class of 2015 (*F* (1, 71) = [4.18], *p* = 0.044, 95% CI [108.55, 115.63]), and the Class of 2019 (*F* (1, 102) = [4.38], *p* = 0.039, 95% CI [116.73, 120.79]). When examining all cohorts combined, females also had significantly higher empathy scores than males (*F* (1, 489) = [9.58], *p* = 0.002, 95% CI [110.77, 113.28]). Refer to Table 4.

**Table 4:** Means and Standard Deviations of JSPE-S Scores of 493 Medical Students at Five Time Points (Time 1-Time 5)^a^ by Class Cohort and by Gender

Class Cohort	Gender	Time 1		Time 2		Time 3		Time 4		Time 5	
		No.	Mean (SD)	No.	Mean (SD)	No.	Mean (SD)	No.	Mean (SD)	No.	Mean (SD)
2014	Male	29	106.3 (11.9)	22	111.5 (13.2)	28	104.2 (10.9)	29	96.3 (15.9)	16	107.5 (10.6)
	Female	23	115.0 (9.2)*	20	114.5 (9.1)	24	97.3 (15.7)	22	101.1 (12.8)	16	106.2 (11.9)
2015	Male	39	105.7 (15.5)	34	100.9 (12.6)	36	106.3 (12.5)	NA	NA	33	110.9 (13.5)
	Female	34	112.1 (10.1)*	35	99.4 (12.4)	33	103.2 (14.2)	NA	NA	33	110.7 (13.7)
2016	Male	38	100.4 (8.1)	35	103.9 (12.7)	34	113.2 (12.7)	30	113.9 (12.1)	36	111.2 (15.2)
	Female	30	103.3 (7.3)	32	107.9 (10.2)	31	115.1 (8.1)	29	115.7 (12.02)	31	117.0 (10.8)
2017	Male	62	108.7 (6.3)	62	111.0 (12.8)	58	113.0 (14.8)	43	112.1 (11.8)	52	111.9 (15.3)
	Female	34	107.7 (6.3)	34	117.1 (9.5)	34	116.9 (11.9)	26	118.0 (12.3)	34	117.9 (11.9)
2018	Male	43	112.7 (10.6)	43	108.8 (12.4)	34	111.4 (11.7)	35	106.5 (14.0)	36	107.7 (12.0)
	Female	55	113.7 (8.4)	51	110.2 (10.4)	50	114.1 (8.4)	45	110.9 (10.9)	45	114.1 (9.7)*
2019	Male	67	115.2 (9.2)	62	112.5 (10.9)	56	112.4 (13.9)	59	109.4 (13.7)	34	112.2 (13.3)
	Female	37	118.8 (6.1)*	34	115.9 (8.3)	32	115.0 (10.1)	34	114.0 (9.7)	18	117.6 (12.8)
All Cohorts	Male	278	109.1 (11.2)	258	108.8 (12.8)	246	110.7 (13.4)	196	108.2 (14.4)	207	110.6 (13.8)
	Female	213	112.0 (9.3)*	206	110.5 (11.7)	204	111.1 (13.1)	156	112.3 (12.4)	177	114.3 (12.0)

The table shows results of an ANOVA comparing baseline scores across cohorts by gender

*No.* number; *SD* standard deviation; *NA* not analyzed

*Females’ empathy scores were significantly higher at noted time point and cohort; *p* < 0.05

^a^The JSPE-S was administered to students at the beginning of each academic program year (Times 1–4) and at time of graduation (Time 5)

### Empathy at End of Program

The overall effect of time on JSPE-S scores was significant (*p* < 0.001); yet, results indicate that mean empathy scores for all cohorts combined (Classes of 2014-2019; N = 493) did not change by the end of the program, compared to the beginning of the program (*p* = 0.08, 95% CI [− 5.88, 0.38]). Mean JSPE-S scores and standard deviations for all cohorts, years, and time points are presented in Table 2. In addition, the overall effects of class cohort and gender were statistically significant at *p*< 0.001, respectively. Table 5 reports the overall effect of JSPE-S administration for class cohort, time, age, and gender.

**Table 5:** Parameter estimates and *p*-Values from the linear mixed regression model analyses for 493 medical students. The outcome is JSPE-S score (ranging from 20 to 140)

Variable	Parameter estimate	95% CL	*p Value*
Intercept	118.54	113.31, 123.77	< 0.001
*Class cohort*			
2014	− 6.58	− 10.46, − 2.70	0.001*
2015	− 8.24	− 11.74, − 4.74	< 0.001
2016	− 15.49	− 19.05, − 11.93	< 0.001
2017	− 8.25	− 11.49, − 5.02	< 0.001
2018	− 3.84	− 7.06, − 0.61	0.020*
2019	–	[Reference]	–
*Gender*			
0 = Male	− 2.80	− 4.20, − 1.40	< 0.001
1 = Female	–	[Reference]	–
*Time*			
1	–	[Reference]	–
2	− 2.79	− 5.33, − 0.26	0.031*
3	− 3.45	− 6.07, − 0.84	0.009*
4	− 5.42	− 8.01, − 2.84	< 0.001
5	− 2.74	− 5.87, .38	0.085
*Age group (years)*			
1 = < 22	− 0.026	− 5.25, 5.20	0.992
2 = 22–24	− 0.49	− 5.29, 4.30	0.841
3 = 25–27	0.22	− 4.58, 5.02	0.928
4 = 28–30	0.28	− 4.69, 5.26	0.910
5 = 31–33	0.16	− 5.06, 5.38	0.952
6 = 34–36	2.75	− 2.79, 8.30	0.331
7 = > 36	–	[Reference]	–
*Interaction: class cohort × time*			
2014 × Time 1	–	[Reference]	–
2014 × Time 2	5.42	0.90, 9.93	0.019*
2014 × Time 3	− 6.13	− 10.53, − 1.74	0.006*
2014 × Time 4	− 6.64	− 11.03, − 2.24	0.003*
2014 × Time 5	− 1.04	− 6.29, 4.19	0.695
2015 × Time 1	–	[Reference]	–
2015 × Time 2	− 5.62	− 9.56, − 1.68	0.005*
2015 × Time 3	− 0.22	− 4.23, 3.77	0.912
2015 × Time 4	N/A	N/A	N/A
2015 × Time 5	4.47	0.00, 8.95	0.050*
2016 × Time 1	–	[Reference]	–
2016 × Time 2	7.03	3.04, 11.03	0.001*
2016 × Time 3	15.69	11.60, 19.78	< 0.001
2016 × Time 4	17.95	13.76, 22.14	< 0.001
2016 × Time 5	14.57	10.12, 19.02	< 0.001
2017 × Time 1	–	[Reference]	–
2017 × Time 2	7.46	3.73, 11.19	< 0.001
2017 × Time 3	9.28	5.56, 13.01	< 0.001
2017 × Time 4	11.21	7.36, 15.05	< 0.001
2017 × Time 5	8.35	4.25, 12.46	< 0.001
2018 × Time 1	–	[Reference]	–
2018 × Time 2	− 0.84	− 4.47, 2.78	0.647
2018 × Time 3	3.08	− 0.65, 6.82	0.106
2018 × Time 4	1.11	− 2.63, 4.85	0.561
2018 × Time 5	0.741	− 3.40, 4.88	0.726
2019 × Times 1–5	–	[Reference]	–

Linear mixed model analyses were used to assess the interaction of class cohort and time

*CL* confidence levels (lower bound, upper bound); *NA* not analyzed

*p ≤ 0.05

LMM analysis of empathy scores by individual cohorts revealed that JSPE-S scores at the end of the program (Time 5) were either significantly higher (Classes of 2015, 2016, and 2017) or not significantly different (Classes of 2014, 2018, 2019), compared to the beginning of the program (Time 1) (*p* < 0.001). Specifically, for the Class of 2015, scores increased from 108.7 at Time 1 to 110.8 at Time 5 (*p *= 0.05, 95% CI [0.00, 8.95]). For the Class of 2016, scores increased from 101.7 at Time 1 to 113.9 at Time 5 (*p* < 0.001, 95% CI = [10.13, 19.02]). For the Class of 2017, scores increased from 108.2 at Time 1 to 114.2 at Time 5 (*p* < 0.001, 95% CI [4.25, 12.46]). Table 5 includes the interaction effect by class cohort and time of JSPE-S administration.

ANOVAs were conducted to assess Time 5 differences in empathy scores by gender for each cohort. These analyses revealed that empathy scores in females were higher than males only in the Class of 2018 (*F* (1, 79) = [7.09], *p* = 0.009, 95% CI [111.18, 117.00]). When examining all cohorts combined, however, females had higher empathy scores (*F* (1, 382) = [7.09], *p* = 0.005, 95% CI [112.56, 116.13]). Results of this analysis are included in Table 4.

### Empathy trajectories across program years

Scores for the Class of 2014 significantly increased at Time 2 (*p*= 0.019, 95% CI [0.91, 9.94]) and significantly decreased at Times 3 (*p* = 0.006, 95% CI [− 10.53, − 1.74]) and Time 4 (*p* = 0.003, 95% CI [− 11.03, − 2.24]), in comparison to Time 1. However, scores increased by Time 5, resulting in no significant difference between Time 5 and Time 1. For the Class of 2015 scores significantly decreased at Time 2 (*p* = 0.005, 95% CI [− 9.56, − 1.69]) and significantly increased at Time 5, resulting in a higher score at Time 5 compared to Time 1. For the Classes of 2016 and 2017 scores significantly increased at all time points, in comparison to Time 1, with the exception of Time 2, in which the increase in scores for both classes were not statistically significant. For the Classes of 2018 and 2019 scores were significantly lower at all time points compared to Time 1, with the exception of Time 5, at which point scores were not significantly different from Time 1.

**Fig. 3:**
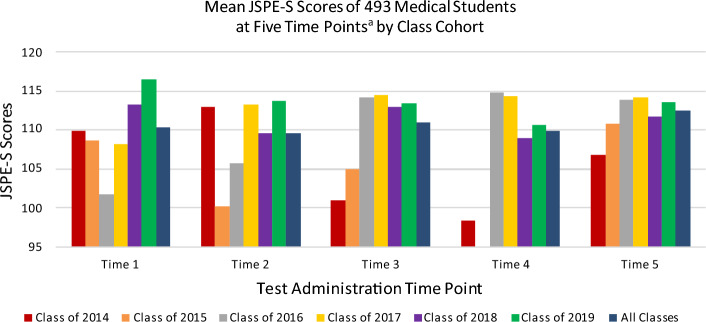
Empathy Scores at Five Time Points by Class Cohort. Note: The figure shows mean empathy (JSPE-S) scores of each class cohort at five time points across the medical education program. ^a^The JSPE-S was administered to students at the beginning of each academic program year (Times 1–4) and at time of graduation (Time 5). Time 4 data is not available for Class of 2015

For the combined cohorts, scores significantly decreased at Time 2 (*p* = 0.031, 95% CI [− 5.33, − .26]), significantly increased at Time 3 (*p* = 0.009, 95% CI [− 6.07, − .85]), and significantly decreased at Time 4 (*p* < 0.001, 95% CI [− 8.01, − 2.84]) in comparison to Time 1. However, scores increased by Time 5, resulting in no significant difference between scores for combined cohorts at Time 5, compared to Time 1. Table 2 outlines the mean JSPE-S score at each time point by class cohort, and the interactions of cohort and time the JSPE-S was taken. Figure 3 provides a graphical depiction of empathy trajectories by class cohort across time points.

## Discussion

Our study is unique in two aspects. First, we examined empathy in medical students progressing through an integrated curriculum, where patient contact occurs early and is embedded throughout. This is unique among empathy studies, especially those conducted in the U.S., which typically describe a traditional 2 × 2 curriculum, where patient contact starts in Year Three of the four-year program. Second, given the reported lack of longitudinal, time-series research designs, we collected data from six student cohorts, assessing empathy at five time points during students’ progression in the program.

We found that students’ empathy levels were either higher or not different at the end of the medical education program. Of the six student cohorts included in our analysis, three cohorts exhibited significantly higher empathy levels at the end of the program and three cohorts’ empathy levels were not significantly different at the end of the program, compared to the beginning of the program. Therefore, none of the six cohorts indicated a decline in empathy at the end of the program. Analyzing all cohorts combined, we found empathy levels by the end of the program were not significantly different from the beginning of the program. These results confirmed our hypothesis of no decline in empathy at the end of the medical education program.

This finding is consistent with recent reports of stable or increased empathy levels (Costa et al.^2013^; Kataoka et al., 2009; Magalhães et al., 2011; Roh et al., 2010; Wen et al., 2013). Although these studies were conducted outside of the U.S., results of non-declining empathy in medical students challenge earlier reports of empathy declines and support the notion of context specificity, suggesting certain learning contexts may result in non-declining empathy patterns (Eva, 2003; Quince et al., 2016). Our findings of stable or increased empathy levels at program end add to this body of research and underscore the need to further examine empathy within differing educational settings and curricula.

Looking specifically at empathy levels in Years Three and Four, our results partially confirmed our hypothesis of no decline in these program years. In two cohorts (Class of 2016 and 2017) we found empathy levels to be significantly higher at the beginning of Year Three and Year Four. In addition, one cohort (Class of 2015) demonstrated empathy levels in Year Three that were not different from Year One (data for Year Four was not available for this cohort). These results confirmed our hypothesis. However, in three other cohorts (Classes of 2014, 2018, and 2019) we found significant decreases in empathy levels at the beginning of Year Three and Year Four, when compared to the beginning of the program. While this finding is consistent with reports of declining empathy levels among U.S. medical students (Austin et al., 2007; Bellini & Shea, 2005; Chen et al., 2007, 2012; Hojat et al., 2004, 2009; Neumann et al., 2011; Newton et al., 2008), all three cohorts’ scores increased by the end of Year Four, such that empathy levels at the end of the program were not different compared to the beginning of the program.

Our analyses also revealed significant decreases in empathy as early as Year Two for three individual cohorts (Classes of 2015, 2018 and 2019) and for the combined cohorts. While we did not expect this outcome, it is consistent with recent studies reporting early declines in empathy (Chatterjee et al. 2017; Newton et al., 2008; Nunes et al., 2011), and could suggest that students experience reactions to the realities of patient relationships sooner, given the early introduction of patient interactions. Patterns of early decline have also been discussed as a “settling in phenomenon with a change from idealism to realism”, with students “displaying an adaptive response to new responsibilities and an increasing workload” (Nunes et al., 2011, p. 12). In addition, Chatterjee et al. (2017), reported empathy levels to decline initially and then rebound over time. This is the pattern we observed in all cohorts exhibiting an early decline in Year Two, as these cohorts demonstrated stable or increased empathy levels by the end of the program, in comparison to program start. Differences in research designs make it difficult to conclusively interpret previous findings alongside ours. For example, Nunes et al., (2011, p.17) utilized a cross-sectional design, noting “a repeated measures design would have been more logical”. In general, a lack of multi-cohort, longitudinal, time-series studies on empathy makes it challenging to interpret the variability we found between our student cohorts and warrants further examination of local contextual factors that may have impacted these outcomes.

Another important finding of our study is the variability in empathy patterns among individual cohorts across time points. While some cohorts demonstrated fluctuating or decreasing empathy levels across program years, two cohorts—the Classes of 2016 and 2017—exhibited increases at all time points. Review of empathy at baseline reveals these two cohorts had the lowest mean empathy levels at the onset of the program among all cohorts. Therefore, it is difficult to make any statements regarding trajectories in relation to baseline levels. We plan to conduct in-depth analyses of cohort characteristics and program variables to better understand the factors that may have led to the variability we found among cohorts.

Despite variation in trajectories across years, mean empathy scores for all individual cohorts and combined cohorts were either not significantly different or significantly higher by the end of the program, demonstrating that empathy can be stable or increased at the end of a program, even if levels decreased or fluctuated over the course of the program. Furthermore, our results provide evidence that empathy does not need to decline among U.S. students and challenges the suggestion of geo-sociocultural patterns (Ponnamperuma et al., 2019). It needs to be noted that most U.S. studies reporting empathy declines in medical students are not recent, emphasizing the need to re-examine medical student empathy in U.S. medical school programs, especially those with reformed curricula.

Based on our analyses, we cannot determine whether our curricular structure and its embedded components directly counter the “emotional detachment, affective distance and clinical neutrality” that has been described to occur in traditional medical education (Hojat et al., 2009, p. 1188). However, the finding that none of our six class cohorts exhibited a decline in empathy at the end of the four-year program supports the notion that empathy might be positively impacted by certain curricular structures.

Our medical education program integrates basic and clinical sciences, includes early patient experiences, and embeds patient-centered learning activities throughout all four years of training. In addition, the program’s emphasis on Medical Spanish, cultural competency, and regular reflection through seminars and writing, aligns with recent research discussing the potential positive impacts of students’ sensibility to language, interactions, communication, active listening, and reflective writing on empathy (Krishnasamy et al., 2019). We plan to further examine the potential effects of our program’s curricular elements on empathy in future analyses. Overall, our outcomes emphasize the need to analyze integrated curricula in medical education, and the nature of early patient interactions within these specific learning contexts, to better understand their impacts on student empathy.

### Limitations

Our findings need to be interpreted with consideration of the following limitations. The JSPE-S is a self-report measure and a reflection of students’ ideals, which may not translate into medical practice. One report suggests scale language might impact results, noting that the English version JSPE-S used in the U.S. has consistently failed to produce significant positive changes, compared to translated versions (Ponnamperuma et al., 2019). Our results directly contradict this proposition, while supporting the conception that contextual differences impact emerging trends in empathy patterns. In addition, the JSPE-S is structurally different from the IRI, the second most used instrument in studies of empathy. Therefore, results of studies using the JSPE-S may not be comparable with studies using the IRI (Quince et al., 2016).

Located in a majority Hispanic community that is also medically underserved, the mission of our medical school includes a focus on underrepresented populations and service to the local community. As such, it is possible that the school attracts individuals with certain inherent values and ideologies. Faculty conducting applicant interviews and the make-up of the admissions committee also change over time, which could have effects on incoming cohort characteristics and in turn impact empathy self-assessments. On the other hand, our analysis shows only one cohort differing significantly in baseline empathy, with a significantly lower JSPE-S score (101.2) at the beginning of the program. The remaining cohorts’ mean scores ranged from 108.2 to 116.5. Unfortunately, a norm table for students of allopathic medicine programs is currently not available (Hojat et al., 2011). However, Hojat (2016, p. 124) notes “in national and international studies, the reported JSE mean scores vary, mostly hovering around 112”. Therefore, cohort baseline JSPE-S scores appear to be within a typical range.

Certainly dynamics that develop within groups, based on individual and group traits, as well as time-based social and political contexts, might influence feelings of empathy as students mature through the program. Future analysis of student characteristics and differences among cohorts should provide more answers to what might have contributed to variations in trajectories.

Lastly, the fact that our sample is from a single medical school is also a limitation. Although, the school is similar to other U.S. medical schools in regards to class size, graduation rates, and residency match results. In addition, given that all students in our medical education program are required to follow a prescribed course of study, we could not incorporate a control group into our research design. This is a dilemma educational researchers often face, since program delivery cannot be altered for research purposes. For comparisons, we provided an extensive review of the literature, covering several decades of research on empathy in medical students, including research that has been conducted in varying curricular environments across countries.

To contextualize our study environment, we provided background on the medical education reform in the U.S. leading to adoption of the integrated curriculum, and we provided a description of the integration of basic and clinical sciences at our medical school. Generalization of our findings can be enhanced by replicating this study at medical schools with similar curricular structures in the U.S. and abroad. Despite these limitations, we believe our findings make an important contribution to the literature, since most cases of stabilizing or increasing empathy levels have been reported outside of the U.S.

## Conclusions

Our study is significant in several aspects: It provides much needed data on medical student empathy by mapping empathy trajectories over the entire course of a medical education program, as well as longitudinally across six student cohorts. Our findings indicate that empathy trajectories do not result in a decline of empathy at the end of the medical education program. In addition, results from our analyses of combined cohorts do not support reported patterns of empathy decline during the third and fourth year in U.S. programs. Our data also shows variation of empathy trajectories between cohorts over the course of our study period. Nonetheless, empathy levels for all six cohorts were either statistically unchanged or higher at the end of the program, compared to the beginning of the program. The fact that some cohorts experienced declines, which later stabilized or increased, suggests empathy levels can improve over time and, generally, might vary more across the length of a program than previously thought. Future research should avoid the assumption that empathy remains low after a decline and should include multiple time points to measure empathy as educational programming and training progresses.

Overall, our results provide support for the notion that student empathy levels do not need to decline during medical training, and that these outcomes can occur in a U.S. medical school environment that delivers an integrated curriculum. As such, our study supports the concept that student empathy trajectories might differ as a result of school curricular structures and approaches to medical education and training. While the parameters of our study do not allow for definitive conclusions regarding the direct impact of integrated curricula and early-patient contact on students’ empathy trajectories, our findings suggest that introducing students to patients early and throughout their training may play a part in establishing an overall empathy-protective, possibly empathy-enhancing learning and training environment.

Continued assessment of student-patient interactions and their impact on student empathy, as well as other potential contributing factors to fluctuations in empathy across time points is warranted. We recommend that future studies examine empathy longitudinally, employing repeated measures designs, within various learning contexts. We further recommend specifically analyzing the impact of the timing, quantity, and nature of student-patient interactions and patient-centered learning activities on students’ empathy trajectories during education and training.

## Data Availability

The data that support the findings of this study are available from the corresponding author, [C.H.V.], upon reasonable request.
